# Resident Physician Attitudes and Competence About Obesity Treatment: Need for Improved Education

**DOI:** 10.3885/meo.2008.Res00257

**Published:** 2008-05-02

**Authors:** Nichola J. Davis, Himani Shishodia, Bizath Taqui, Claudia Dumfeh, Judith Wylie-Rosett

**Affiliations:** † St. Luke's Roosevelt, New York, New York, USA; * Temple University Hospital, Philadelphia, Pennsylvania, USA; § Albert Einstein College of Medicine, Montefiore Medical Center, Bronx, New York, USA

**Keywords:** obesity, resident education, treatment

## Abstract

**Background:**

Obesity is a common problem in primary care, but little is known about Internal Medicine residents’ attitudes towards obesity treatment.

**Objective:**

To describe resident attitudes about obesity treatment.

**Methods:**

Cross-sectional survey of 101 Internal Medicine residents in Philadelphia, PA, and Bronx, NY. Responses to 18 items on a Likert scale assessed resident attitudes. Weight loss goals were assessed with open-ended questions to a clinical scenario. ANOVA with trend analysis compared questionnaire responses to resident postgraduate year (PGY) level. Associations between clinic site, PGY level, and dichotomized Likert responses were tested with chi-square analysis.

**Results:**

19% of residents felt competent in prescribing weight loss programs. Few residents (18%) considered the current recommendations of a 5-10% reduction in body weight to be successful in an obese hypothetical patient. Third-year residents reported greater feelings of negativity towards obese patients than first- and second year residents (p<.05)

**Conclusions:**

Resident physicians do not feel competent in treating obesity and have unrealistic weight loss goals; third-year residents had more negative attitudes about obese patients compared to residents in their 1^st^ or 2^nd^ year of training. These areas are targets for further resident education about obesity management.

There is a global epidemic of obesity. Primary care physicians are integral in treating obesity, yet physicians offer weight-loss advice to a minority of obese patients seeking primary care.^1^ Prior studies have demonstrated that physicians lack confidence in treating obesity and may have negative attitudes towards obese patients.^2^,^3^ Understanding the attitudes of physicians-in-training (residents) can inform educators about areas for further curriculum development. Additionally, increasing resident awareness about these attitudes may affect their management of obese patients. This study describes resident physicians’ attitudes about obesity treatment and identifies areas that can be addressed in resident education.

## Methods

**Setting** - We conducted a cross-sectional survey of Internal Medicine residents at Temple University in Philadelphia, PA, and Montefiore Medical Center in Bronx, NY. Residents attending Internal Medicine clinic between August 2005 and January 2006 were invited to participate. Surveys were distributed to residents at the beginning of their clinic session and collected the same day. There were no incentives for participation at either site. The study was approved by the Internal Review Boards of Montefiore Medical Center and of Temple University.

**Survey Instrument** - We adapted a questionnaire previously used to assess practicing physicians’ attitudes 4 and abbreviated it for ease of completion during the clinic session. The original questionnaire addressed 5 different domains including causes of obesity, attributes of obese individuals, beliefs about obesity treatment, weight loss outcomes, and relative efficacy of obesity treatment. For the present study, we used three domains from the original survey: causes of obesity, beliefs about obesity treatment, and weight loss outcomes without any additional modification. The survey was used with permission from the authors.^5^ The survey consisted of eighteen items (Table 1) rated on a 5-point Likert scale ranging from “1 = strongly disagree” to “5 = strongly agree” to assess resident attitudes. One open-ended question presented a clinical scenario of a hypothetical female patient with type-2 diabetes who weighed 200 pounds and was 5'5” in height. (Body Mass Index =33kg/m2). Residents were asked to report the weight loss outcome that they would consider successful.

**Table 1:** Resident Attitudes and Knowledge of Obesity Care, A Cross-Sectional Survey

Survey Items	Agree/Strongly Agree(%)			
	PGY1	PGY2	PGY3	p value
1. Obesity is a chronic disease.	91	94	96	.63
2. Obese patients could reach a normal weight if they were motivated .	51	47	58	.96
3. I have negative reactions towards the appearance of obese patients.	17	24	54	.001
4. I believe it's necessary to educate obese patients on the health risks of obesity.	97	97	100	.41
5. For most obese patients, long term maintenance of weight loss is impossible.	14	19	19	.45
6. Most obese patients are well aware of the health risks of obesity.	29	22	15	.09
7. If a patient meets the appropriate criteria I would recommend an evaluation by a surgeon.	51	62	58	.80
8. I often feel uncomfortable when examining an obese patient.	6	11	11	.66
9. Obesity is associated with serious medical conditions.	94	100	100	.25
10. I am usually successful in helping obese patients lose weight.	11	11	8	.28
11. Medications used to treat obesity should be used chronically.	14	11	4	.77
12. Most obese patients will not lose a significant amount of weight.	47	35	73	.006
13. It is acceptable to apply “scare tactics” to obtain the compliance of the obese patient.	24	43	50	.14
14. It is difficult for me to feel empathy for an obese patient.	3	11	15	.11
15. I feel competent in prescribing weight loss programs for obese patients.	14	27	15	.20
16. A 10% reduction in body weight is sufficient to significantly improve health complications.	54	62	50	.71
17. Physicians should be role models by maintaining a normal weight.	80	76	96	.18
18. Medications used to treat obesity should be limited to short-term (<3 months) use.	47	49	58	.76

**Statistical Analysis** - One-way ANOVA with trend analysis compared mean questionnaire responses by resident PGY (postgraduate year) level. Likert scale responses were dichotomized by collapsing strongly agree and agree into one category, “agree”. Strongly disagree, disagree, and neutral were collapsed into the second category, “disagree”. Chi-square analysis tested associations between dichotomized responses, clinic site, and PGY level. A p value less than 0.05 defined a statistically significant difference or association.

## Results

**Resident Characteristics** - Of 156 residents invited to participate, 101 (65%) responded. Respondents included 71 residents from Temple and 30 from Montefiore Medical Center. Resident demographics and questionnaire responses did not differ between sites. Respondents were 55% male and distributed across training levels with 35% PGY1s, 37% PGY2s, and 27% PGY3s. Data on PGY category were missing for two respondents.

**Resident Attitudes about Obesity Treatment** - More than 90% of residents agreed that obesity (Body Mass Index >30kg/m2) is a chronic disease, that it is necessary to educate obese patients about health risks, and that obesity is associated with serious medical conditions. Half of the respondents believed that obese patients could reach a normal weight if they were motivated, and 57% of the residents agreed that a 10% reduction in body weight was sufficient to improve health-related outcomes. Few resident respondents (19%) felt competent in prescribing weight-loss programs, and 10% agreed that they were successful in helping patients lose weight. Few residents (8%) reported feeling uncomfortable examining obese patients or difficulty feeling empathy for obese patients.

Most PGY3s (72%), compared to 47% of PGY1s and 35% of PGY2s, agreed that obese patients will not lose a significant amount of weight (difference in proportions statistically significant at p = 0.006). Additionally, 56% of PGY3s reported feelings of negativity towards the appearance of obese patients, compared to 17% of PGY1s and 24% of PGY2s (p = 0.001). Twenty-seven percent of PGY2 residents reported feeling competent in making weight-loss recommendations compared to 14% of PGY1s and 15% of PGY3s. This difference was not statistically significant (p=.20).

Goals of weight loss treatment-For the clinical scenario, we defined a weight loss of 5-10% as a successful outcome.^6^ Only 18% of resident respondents reported a weight loss within this range as successful, while 82% of residents reported a weight loss greater than 10% as successful. The mean weight-loss outcome reported as successful was equivalent to a 21% weight reduction, a finding that did not differ between PGY levels (p = 0.19).

## Discussion

Our study demonstrates that while resident physicians recognize obesity as a serious medical condition, most feel incompetent in treating obesity and have unrealistic weight loss goals; residents in their third year of training have more negative reactions to obese patients than residents in other years. These findings highlight areas that may benefit from further emphasis in residency training programs.

Prior studies of Internal Medicine residents demonstrated that a minority of residents felt qualified in treating obesity.^7^ This study's findings were similar in that most residents did not feel competent in treating obesity and reported little success in treating obesity. Interestingly, third-year residents did not feel any more competent in treating obesity than first- or second-year residents, further emphasizing the need for education about obesity management during residency training. Physicians in practice tend to report higher levels of competence which may reflect experiences in clinical practice.^2^

Our study further highlights the unrealistic weight loss goals held by residents. While many residents agreed in theory that a 10% reduction in body weight was sufficient to improve health-related outcomes, when presented with a hypothetical clinical scenario most residents cited weight loss goals exceeding 10% as a successful outcome. It is well known that patients have unrealistic weight loss goals that may lead to frustration and disappointment with weight-loss attempts.^5^ Similarly, among resident physicians unrealistic goals may contribute to resident frustration and feelings of futility when modest reductions are not recognized as successful. Resident education, therefore, needs to focus on establishing realistic goals and on working with obese patients to develop strategies to achieve these goals.

Compared to 1st- and 2nd- year residents, 3rd- year residents reported more negative reactions to the appearance of obese patients. It is important to recognize the potential effect that this may have on the treatment of obese patients. Several studies indicate that healthcare professionals possess explicit negative attitudes about obese patients, including viewing them as unattractive, noncompliant, and lazy.^8^ Unfortunately, these negative attitudes may affect the medical care received by obese patients. One survey of obese patients reported that doctors were a significant source of weight stigma experienced by overweight and obese patients.^3^ Our study did not ask about specific negative attitudes. Because of its cross-sectional nature, we cannot determine if the negative reactions reported by third-year residents increased during residency training. It is important, however, to recognize that negative attitudes may exist among residents and that addressing these attitudes is part of the challenge in educating residents about obesity.

Education of residents should additionally involve teaching residents concepts of chronic illness management that promote collaborative interactions between physician and patients with the use of brief interventions such as WAVE (Weight, Activity, Variety, Excess screener) and REAP (Rapid Eating Assessment for Patients) that can be successful tools in primary care settings. 9 There is increasing evidence that patient self-management is essential to improving outcomes in chronic diseases, including obesity^10^,^11^ and that this is another skill set that resident physicians would benefit from learning. Additionally, it is important to note that successful obesity treatment is often accomplished through intensive interventions with frequent follow-up with the medical provider. Residents can play a key role in the coordinated care of obese patients by utilizing additional services such as those of dietitians and psychologists when available.

Based on our study findings, we have developed an educational intervention focused on assessment and management of obesity which is being implemented and tested at one study site.

**Limitations** - The generalizability of our findings are limited due to the low response rate. In addition, there is evidence in the literature of differences in physician attitude and recommendations to patients based on patient gender. We could not evaluate the role of patient gender in this study since our clinical scenario used only a female patient.^12^ Finally, the cross-sectional study design prevented us from determining if the observed differences in resident attitudes between PGY level were the results of changes in attitude that occurred during residency training.

## Conclusions

Resident physicians in our study at all levels of training did not feel competent in treating obesity and had unrealistic weight loss goals. These areas may be improved with resident education. The differences in negative reactions to obese patients between PGY levels is intriguing and will require further study to determine if these attitudes develop over time and what factors contribute to these negative attitudes.

## Conflict of Interest

The authors report no conflict of interest.
